# Exceptional longevity in northern peripheral populations of Wels catfish (*Silurus glanis*)

**DOI:** 10.1038/s41598-022-12165-w

**Published:** 2022-05-16

**Authors:** Kristofer Bergström, Oscar Nordahl, Peter Söderling, Per Koch-Schmidt, Tobias Borger, Petter Tibblin, Per Larsson

**Affiliations:** 1 Department of Biology and Environmental Science, Centre for Ecology and Evolution in Microbial Model Systems EEMiS, Linnaeus University, 391 82 Kalmar, Sweden; 2 Fish and Wildlife, The County Administrative Board of Kalmar County, Regeringsgatan 1, 391 86 Kalmar, Sweden

**Keywords:** Ecology, Zoology

## Abstract

Studies of life-history variation across a species range are crucial for ecological understanding and successful conservation. Here, we examined the growth and age of Wels catfish (*Silurus glanis*) in Sweden, which represent the northernmost populations in Europe. A total of 1183 individuals were captured, marked and released between 2006 and 2020. Mark-recapture data from 162 individuals (size range: 13–195 cm) were used to estimate von Bertalanffy growth curve parameters which revealed very slow growth rates compared to catfish within the core distribution area (central Europe). The fitted von Bertalanffy growth curve predicted a 150 cm catfish to be around 40 years old, while the largest recaptured individual (length 195 cm) was estimated to be 70 (95% CI 50–112) years old. This was substantially older than the previously documented maximum age of a catfish. The weight at length relationships in these northern peripheral populations were similar to those documented for catfish in central Europe indicating that resources did not constrain growth. This indicates that the slow growth and exceptional high age in the northern catfish populations are the result of lower temperatures and/or local adaptations.

## Introduction

Longevity (lifespan) is an important component of an organism’s life-history and is inherently connected to body size, growth rates and age-at-maturity, especially in species with indeterminate growth such as fish^1–3^. The longevity (survival) of individuals can also be influential to the viability and dynamics of populations, particularly in iteroparous species inhabiting environments that only sporadically present suitable environmental conditions for reproductive success^4–6^. As such, longevity is a crucial factor to consider in assessments of the viability of populations and if they are vulnerable to exploitation^5,7^.

Thermal conditions and resource availability can modulate growth rates and longevity by influencing metabolic rates and senescence^8,9^. For instance, lower temperatures and/or diet restrictions often results in decreased growth rates, later maturation and increased longevity^10,11^. Differences in longevity among populations may also reflect local adaptations in growth and age-at-maturity, with a well-documented pattern of initial slow growth and late maturity connected to higher longevity and *vice versa*^3,11^. Comparisons of longevity along latitudinal (thermal) gradients have contributed important ecological insights including evaluation of the temperature-size rule and temperature-constraint hypothesis^11,12^. In this context, further insight into the role of environmental variation in structuring longevity can be gained by studying longevity and growth rates in climatic peripheral populations.

It is challenging to study growth trajectories and longevity in large and long-lived organisms where it is not feasible to follow their complete life-cycles and cannot be sacrificed, to use calciferous structures or similar to estimate age and growth, due to being protected. In this context, mark-recapture studies have been proven to be a valuable and robust method, for example in fish species as catfish and sturgeon^13–16^. However, to obtain robust estimates of growth trajectories and age, this method requires recapture across multiple years of large numbers of marked individuals that comprises the complete size structure. This task is not easily undertaken in long-lived apex predators due the inherent constraints of studying large organisms which are often combined with low abundances of individuals in the upper size/age classes.

**Figure 1:**
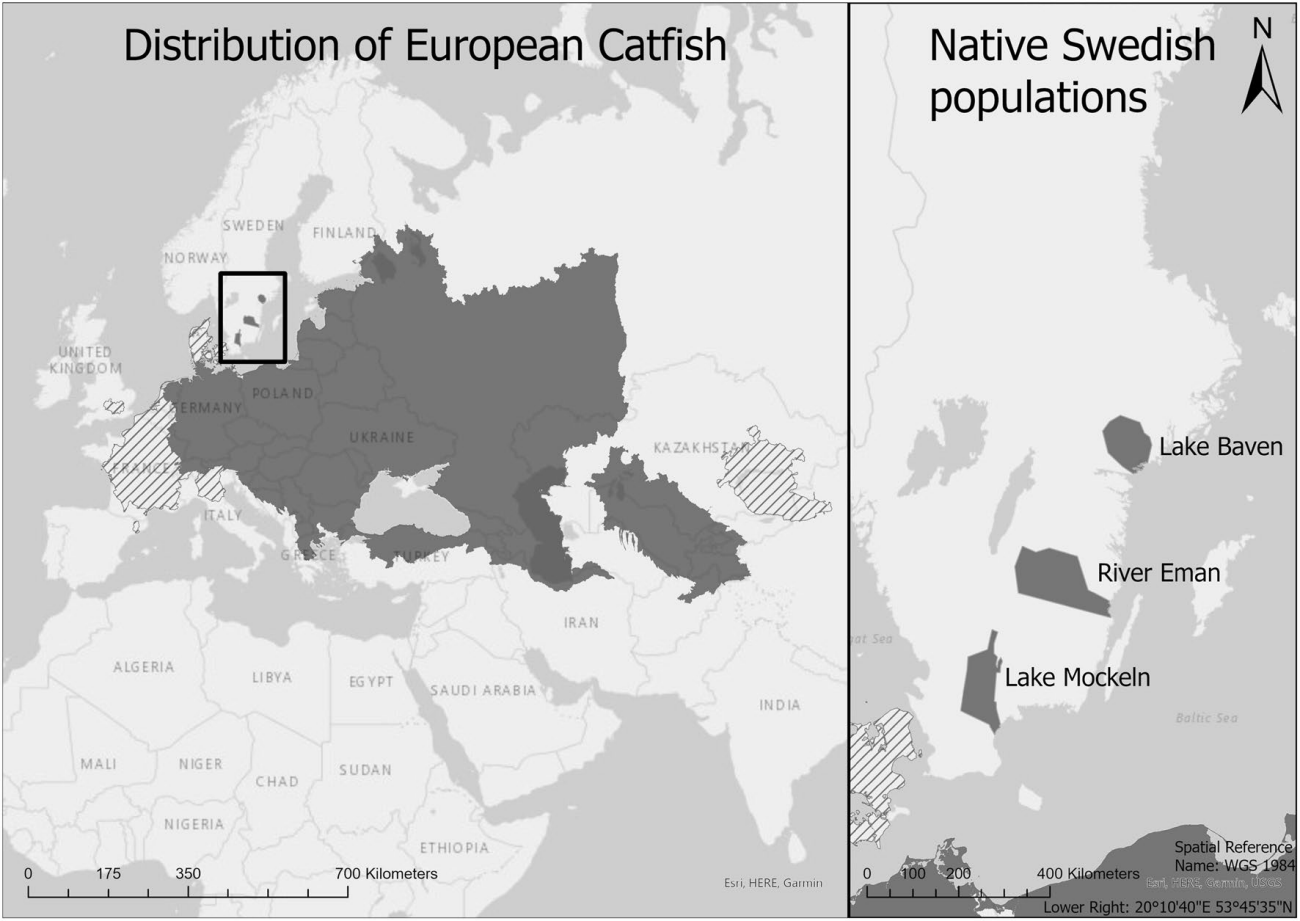
Map showing the distribution of European Catfish, native populations in dark grey and introduced in striped. Redrawn from IUCN^17^, map created in ArcGIS software by Esri (ArcGIS Pro 2.7.1 https://arcgis.com/).

Here, we investigate growth trajectories and longevity in northern peripheral populations of Wels catfish (*Silurus glanis*, hereafter catfish). Catfish are iteroparous, apex and opportunistic predators and are the largest freshwater fish species in Europe whose natural distribution area is in central-eastern Europe and western Asia^17^. To the south, their natural distribution area is limited by a longitudinal line from the River Rhine to the Aral Lake. To the north their natural distribution is limited to three postglacial remnant populations (present in Lake Möckeln, River Emån and Lake Båven) in southern Sweden. Native populations of catfish are extinct elsewhere in Fennoscandia (Fig. 1). Catfish have also been introduced in many aquatic systems in France, Great Britain, Italy and Spain where they are considered an invasive species^18^.

The northern peripheral populations of catfish were established in Sweden during warmer post glacial ages, 9500 to 8000 years ago, when the Baltic Sea was a freshwater lake (the Ancylus Lake). Today, these populations show pronounced genetic differentiation and low allelic diversity compared to the larger populations in central and eastern Europe^19,20^. Microsatellite and single nucleotide polymorphism (SNP) analysis reveals that the Swedish populations not only differ from other European populations, but to a lesser extent also from each other. Moreover, genetic analyses also suggest that the effective population sizes are very low in all Swedish populations^19,20^ which, together with the fragmented and sparse distribution, have contributed to catfish being considered endangered in this peripheral habitat and of high conservation status including complete protection from fishing.

Catfish are a warmwater species with an optimal temperature for growth and reproduction between 25 and 28 °C^18,21^. Growth rates of catfish are rapid in its core distribution area (i.e. central-eastern Europe and western Asia) where thermal conditions are closer to optimal. Previous studies have shown that catfish in these areas can reach a length of 70–130 cm within 5–7 years and become mature at 70–100 cm of length^18,22,23^. Introduced populations further south (e.g. Spain) can reach 150 cm at a similar age^24^. Reviews of growth trajectories suggest that growth in young individuals inhabiting the core distribution area are rapid with annual growth rates of 10–20 cm, whereas after becoming mature (at an age of 4–7 years^18,22,23^) growth rates decrease to 5–7 cm/year^18,23,25^. Estimates of longevity also suggest that catfish in these areas very rarely become older than 20 years, although individuals reaching an age of 33 years have been documented^22^. However, despite the clear relevance for understanding of catfish ecology, there is still no knowledge regarding similar traits in the northern periphery and how they compare to catfish in the core distribution area in central Europe. Such information is important for protection of populations in peripheral habitats.

To investigate growth rates, growth trajectories and longevity in the northern peripheral populations of catfish, we conducted an extensive mark-recapture study across 15 years. Using a combination of fyke-nets and long-lining, we captured and tagged 1183 individuals ranging from 7 to 209 cm in two (out of three) of the Swedish systems inhabited by catfish. By continuous fishing efforts, we recaptured 162 individuals between 10 months and 10 years post tagging. We then used the mark-recapture data to estimate von Bertalanffy growth curve parameters (henceforth VBGC), growth trajectories and longevity. We also estimated the length–weight relationship. Finally, we compared the growth rates, length–weight relationships and longevities in the focal peripheral catfish populations to previously published estimates of these traits in catfish inhabiting the core distribution area in central-east Europe and western Asia, as well as introduced populations in southern Europe.

## Results

**Figure 2:**
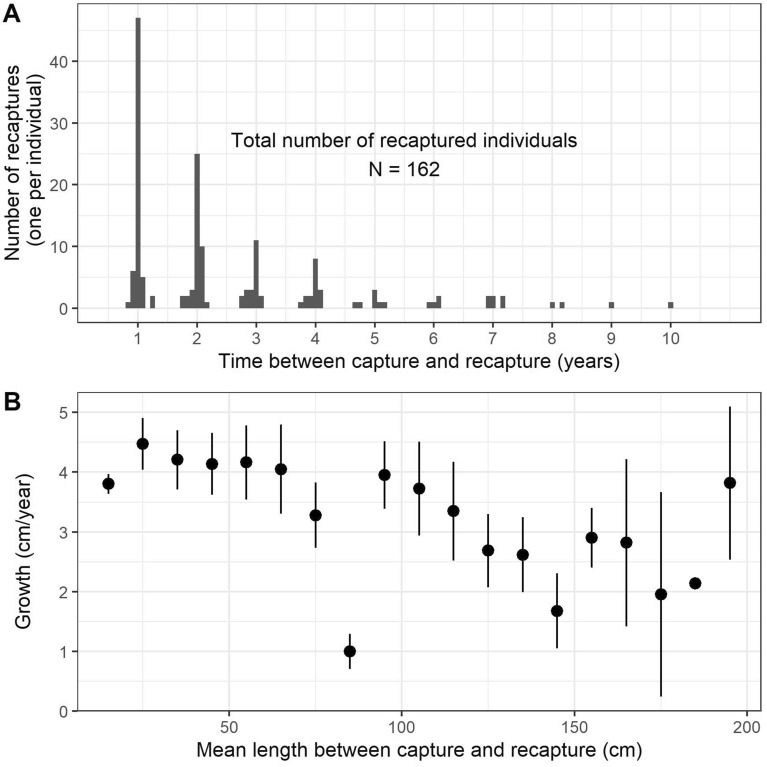
The upper panel (**A**) shows the number of recaptured individuals with time between mark and recapture. Bottom panel (**B**) shows the growth rate (± SE) for the same individuals divided in length size bins of 10 cm.

Out of 1183 captured and marked individuals (*N*_Möckeln_ = 456; *N*_Emån_ = 727), 162 were recaptured within 10 months–10 years from tagging, thereby providing data that could be used to estimate growth trajectories. The mean growth rate (± SE) of recaptured catfish gradually decreased with increasing length from 4.3 ± 0.3 cm/year for fish < 50 cm to 2.8 ± 0.5 cm/year and less for fish > 150 cm (Fig. 2). The growth rate analysis comprised fish with a length at tagging ranging from 13 to 195 cm.

**Figure 3:**
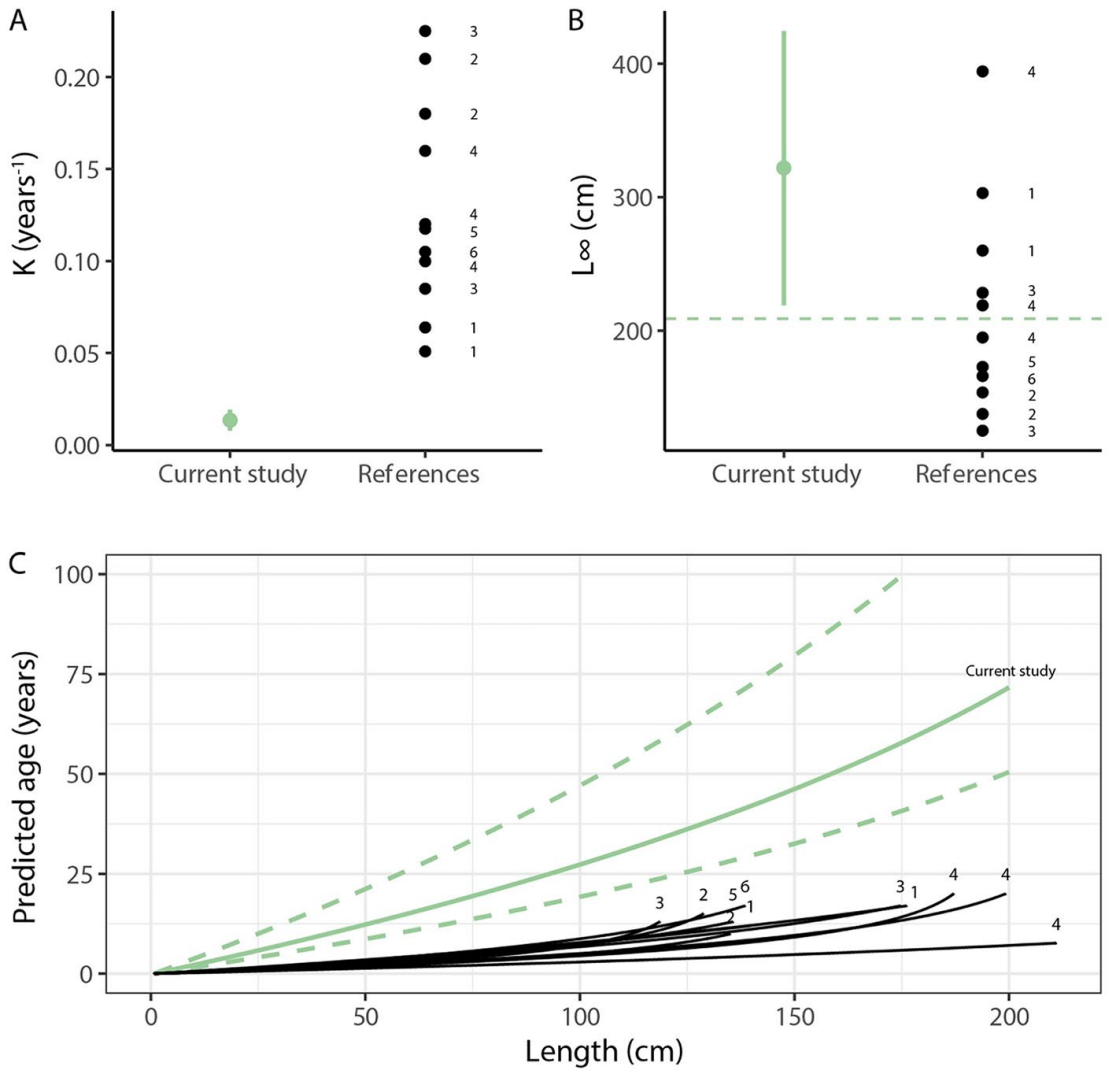
Estimated VBGC parameters K (**A**), L∞ (**B**) and predicted age at length (**C**) for the current study (green) compared to other populations from the core distribution area (black lines and dots; citation for each parameter estimate and growth trajectory: 1^24^, 2^23^, 3^13^, 4^28^ in Ref.^13^, 5^53^, 6^25^). Growth curves for the references were drawn based on values of K and L∞ with the equation Age = loge ((Length − L∞)/(L0 − L∞))/ − K, with L0 set to size at hatching: 0.7 cm^51^. Estimates of K and L∞ are presented along with 95% confidence intervals (green vertical bars). Uncertainty around predicted age (dashed lines) display the 95% confidence interval for K. The dashed horizontal line in (**B**) denote maximum observed length in current study: 209 cm.

The VBGC parameters were estimated to K (± SE, growth): 0.014 ± 0.003 (*t* = 4.706, *P* = 0.000005) and L∞ (± SE, theoretical asymptotic length): 321.7 ± 52.0 (*t* = 6.187, *P* < 0.000000) based on the non-linear regression (RSE = 4.754, df = 160). Predicted age based on VBGC with the estimates of K and L∞ for a fish of 50 cm was about 12 years (95% CI 9–21), and the largest captured individual was about 2 m and estimated to have reached the age of 71 years (95% CI 50–123; Fig. 3). The calculation does not include catfish of 0 + and 1 + ages since these age classes were not captured in this study. The length of 0 + fish has been estimated to 9–12 cm and 1 + to 15–25 cm^26,27^.

The calculated age at length of fish in this study was compared to native populations of catfish in the core distribution area of Europe (Fig. 3). The age of a 2 m catfish from many water courses in Europe is < 20 years^22–24,28^ (Fig. 3). In the Ebro River system catfish at 2 m was found to be about 16 years^24^ and a similar pattern has also been shown in a Turkish resevoir^23^ (Fig. 3). The age of a 2 m catfish at the northern edge of the European distribution range (Sweden) was estimated to be around four times older (~ 71 years). The difference in VB growth trajectory of the Swedish catfish compared to conspecifics in central Europe was not due to a lower asymptotic length (L_∞_ ± 95% CI 322 ± 103 cm), but rather the time it takes (K ± 95% CI 0.013 ± 0.006 years^−1^) for them to reach the asymptotic size (Fig. 3). The oldest previously reported catfish found was from the river Volga in Russia was 33 years and 266 cm long^22^, which is about half the estimated age of our largest recaptured individual at 195 cm.

**Figure 4:**
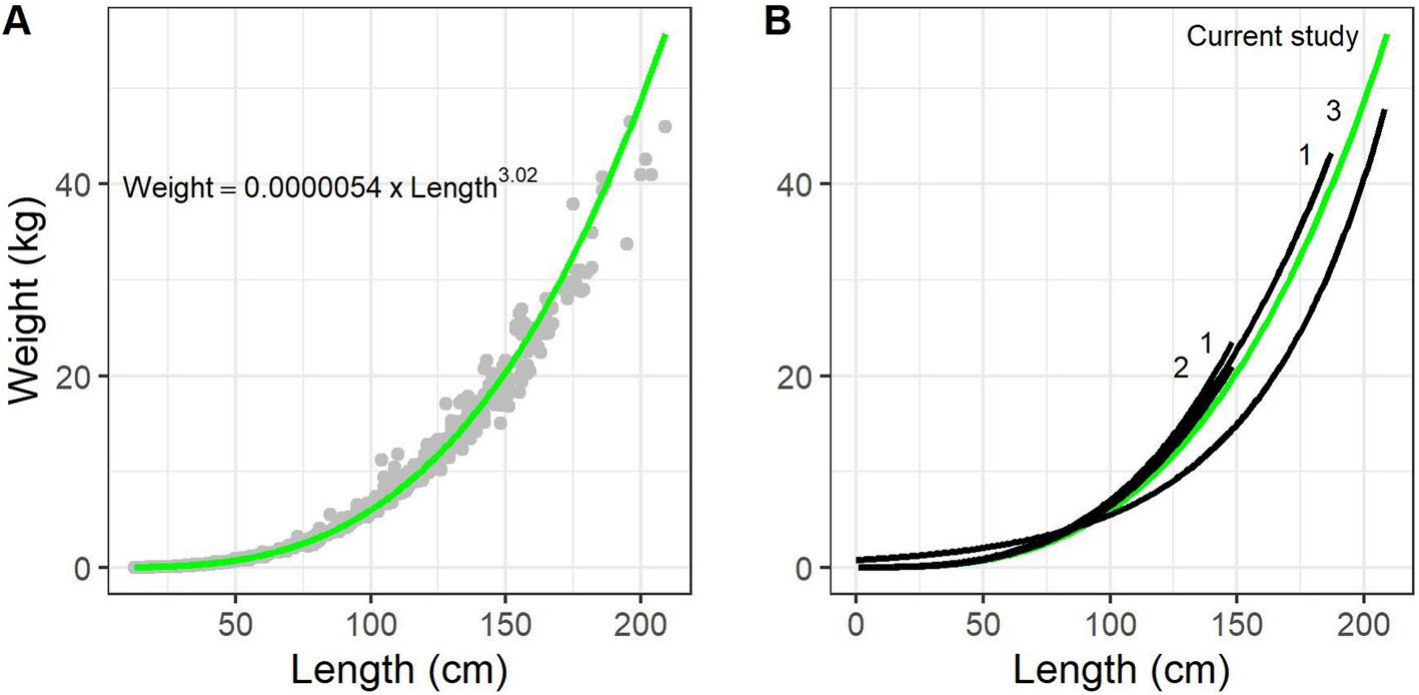
Weight-length relationship for catfish. (**A**) The focal northern peripheral populations. (**B**) Comparison of current study in green with three other reported weight-length relationships from native populations in the core distribution area (1^23^, 2^53^, 3^22^).

The length–weight relationship for Swedish catfish was analyzed by including data from all individuals for which data of weight and length were available (Weight = 0.0000054 × Length^3.02^, *R*^2^ (log–log transformed data) = 0.998, *N* = 937, Fig. 4). Separation by sex was not possible in the analysis since visual sex determination for catfish is challenging outside the reproductive period and in subadults. Individuals with a length of 100 to 150 cm weighed between 6 and 20 kg, while fish less than 80 cm weighed below 5 kg. The longest individual in this study was 209 cm (46 kg, captured in Lake Möckeln) while the heaviest was 46.5 kg (196 cm, captured in River Emån). The length–weight relationship of Swedish catfish was not consistently lower or higher than in the of native populations of catfish from the European core distribution area (Fig. 4).

## Discussion

Long-lived and large organisms have continually fascinated both the public and the scientific society. However, studies of longevity and growth trajectories of such organisms remain challenging and there is still, in most cases, a lack of knowledge regarding whether and how these traits may vary across distribution ranges^29^. We investigated growth trajectories and longevity in Wels catfish, the largest freshwater fish in Europe that can reach a length of more than 250 cm^22^. We performed an extensive mark-recapture study across 15 years in Swedish catfish, which represent the northern peripheral populations, to estimate growth trajectories and longevity and then compared these to corresponding estimates for populations inhabiting the core of the distribution area in central and east Europe. Our results suggest that growth rate was generally very low for individuals in these northern populations in comparison to those from the core habitat, but that there were no differences in the asymptotic/maximum size or the length–weight relationship. Instead, the documented variation in growth rate between core and peripheral populations reflect a pronounced disparity in the shape of the growth trajectory and longevity with individuals in the northern populations becoming profoundly older (large individuals were up to 4 times older compared to similar sized ones in the core of the distribution area). With an estimated age of about 70 years at a size of 200 cm (which is not an uncommon length for catfish to reach throughout its distribution area including Sweden) this substantially exceeds the previously believed maximum age of catfish (approx. 35 years^22^) and render these individuals among the oldest freshwater fish in Europe^30^. This emphasis that peripheral populations may have considerable demographic differences to core habitat populations that must be considered in management and conservation of biodiversity^31,32^. For instance, spurred by the high socioeconomical values of catfish recreational fisheries in central and south Europe (e.g. River Ebro) there is a growing pressure to once again allow angling for Swedish catfish. However, the knowledge of the exceptionally slow growth rate and longevity of these catfish, in combination with their episodic reproductive success, make them vulnerable to exploitation (e.g. angling) that may impair their life-span^5^.

Our results suggest that the growth rates of catfish inhabiting peripheral habitats are comparatively very low throughout their lifespans. In juvenile/subadult stages (< 70 cm), the estimated mean growth rate was near 4 cm/year and already at a sizes above 100–150 cm (and assumed to have reached maturity^18,22,23^) the mean growth rate had decreased to about 3 cm/year. The annual mean growth continued to decrease with individuals at a length of about 175 cm estimated to grow around 2 cm/year. This suggests a strikingly different growth trajectory and age-at-maturity to individuals inhabiting the core distribution area. For instance, a review by Copp et al.^18^ showed annual growth rates of catfish in the European core habitat to range between 5 and 7 cm after maturity was reached at 4–7 years of age and similar patterns have also been demonstrated in populations in eastern parts of distribution area^22,23,33^, as well in introduced populations of catfish in southern Europe^24^.

Interestingly, comparisons of growth trajectories between our peripheral populations to those inhabiting core habitats suggest that the maximum size are similar regardless of area and this is also supported by capture data indicating that the maximum size throughout the distribution area is approximately 2–2.7 m^22,23,34^. Based on the slow growth in the peripheral habitat, this proposes that the major difference between populations is that individuals in peripheral habitats in Sweden become substantially older. For instance, our estimates suggest that individuals in the peripheral populations with a length of around 100 cm fish were about of 25 years old while a 150 cm long fish was about 40 years old which is about four times older than in catfish from the core habitat in central Europe^18,22–24,28^ and constitute among the highest age of freshwater fish known in Europe^30^. Fish becoming older in higher latitudes has previously been shown in several studies^12^ but the magnitude of deviation in age demonstrated here is astonishing even considering the lower bound of the 95% confidence interval. Also increased longevity in high latitude peripheral populations have previously, at least partly, been attributed to differences in asymptotic and maximum size (sensu Bergmann’s rule^35^) rather than slow growth alone^36^.

Theory predicts that there is a growth-longevity trade-off (i.e. slow growth–high longevity and vice versa)^37^ which have been attributed to rates of cell division, oxidative stress and cellular senecence^38^. Previous evidence on this trade-off was largely circumstantial, but recently it was firmly demonstrated that slow-growing phenotypes of three-spined sticklebacks (*Gasterosteus aculeatus*) resulted in a considerably longer (30%) lifespan^39^. The growth-longevity trade-off offers a plausible explanation for our documented pattern but raises the question as to why growth is slow. One putative explanation to the slow growth could be resource limitation^40^, but the similarity to populations in the core habitat in the length–weight relationship argues against. Instead, a more likely explanation would be that catfish in the peripheral habitats experience non-optimal thermal conditions to maximise growth^12^. The optimum temperature for growth in catfish (25–28 °C^18^) very rarely occur in Swedish waters and the duration of suitable temperatures for foraging (i.e. the foraging window), and subsequently growing, will also be constrained by low temperatures during late fall-winter-early spring. For instance, the annual mean water temperature in the focal Swedish systems inhabited by catfish is around 10 °C which is in stark contrast to the higher annual mean temperatures of the core distribution area where catfish grow faster, but have lower maximum ages (e.g. 15.5 °C in river Ebro, Spain^41^ and 13.3 °C in Filyos River in northern Turkey^42^).

Given the above, it is reasonable to assume that the slow growth and high longevity in peripheral populations of catfish are related to phenotypic plasticity mediated by low water temperatures as demonstrated in other species of fish^12,39^. However, it is equally possible that these peripheral populations have evolved local adaptations in growth trajectories and age-at-maturity that impact longevity^1–3^. These populations are geographically isolated and genetically differentiated from the ones in the core distribution area^19,20^ and reside in habitats that only sporadically (i.e. certain years) offer thermal conditions (> 22 °C) that allow for successful reproduction^18,43^. This is also supported by previous genetic studies showing very low effective population sizes in all three peripheral populations of catfish in Sweden^19,20^. Under such circumstances, natural selection should favor a longer reproductive lifespan to maximize the reproductive opportunity^1,2^ but further studies are required to evaluate whether and how peripheral populations of catfish harbor unique adaptive variation that impacts growth rates, age-at-maturity and longevity.

Our results of slow growth and exceptional longevity in catfish inhabiting the northern periphery of its distribution range contribute to advancing the current understanding on the influence of environmental variation on demographic traits. That fish grow slower and may become older in low temperature habitats are not novel per se but our results represent an extreme example on spatial variation in demographic traits despite relatively minor latitudinal variation and should be incorporated into future management and conservation^44^. For instance, slow growing and long-lived fish are very vulnerable to exploitation such as harvest by fisheries^7^. In peripheral habitats which often constitute non-optimal environmental conditions, for instance regarding thermal conditions for reproduction in catfish, harvest of slow growing and old individuals can have dramatic consequences on population dynamics^45^, alter selective regimes^46^ and, ultimately, result in local extinction^44^. In that context, we would like to strongly recommend authorities to maintain the closure of catfish fisheries in Sweden initiated in 1994. Despite a large demand from the recreational fishery to target these giant fish in Sweden once again, more knowledge concerning the dynamics and viability of populations is required for informative management decisions.

To conclude, we show that Wels catfish, the largest freshwater fish species in Europe and an apex predator, inhabiting the northern periphery of its distribution range grows substantially slower and may become much older than conspecifics inhabiting the core habitats in central Europe and western Asia, while maximum size and length–weight relationships were similar. We attribute this to thermal conditions in the peripheral habitats that are likely bordering on what the species can cope with, but whether it reflects local adaptation or phenotypic plasticity remain to be investigated. Regardless of the cause, our study emphasizes that the demography of populations can be vastly different in peripheral habitats also across relatively fine spatial scales which need to be considered in future management and conservation to avoid the loss of biodiversity and negative impacts on ecosystem functioning.

## Methods

### Study populations

Sampling of catfish was conducted from 2014 to 2020 in Lake Möckeln (56° 39′ 48.9′′ N 14° 8′ 57.7′′ E) and between 2006 and 2020 in River Emån (57° 7′ 46.7′′ N 16° 30′ 10.8′′ E) (Fig. 1). Möckeln is a mesotrophic (Tot-P 22.9 ug/L^47^), brown water (130 mg Pt/L^47^) lake with an area of 46.1 km^2^, a mean depth of 2.8 m and a maximal depth of 12 m. The annual mean (1982–2020) temperature is 9.4 °C^47^ and the lake is generally covered by ice in the winter. The fish fauna is representative for lakes in of southern Sweden (with the exception of catfish) with pike (*Esox lucius*), perch (*Perca fluviatilis*) and pikeperch (*Sander lucioperca*) as the dominating predatory species and roach (*Rutilus rutilus*), bream (*Abramis brama*), zope (*Abramis ballerus*) and silverbream (*Blicca bjoerkna*) as the common cyprinids. Emån is a 300 km long river with a mean water discharge of 30 m^3^/s^48^. Catfish only reside in the lower parts (to about 50 km upstream) where the river is mesotrophic (Tot-P 20.3 ug/L^47^) and the water coloured by humic substances (64 mg Pt/L^47^). The annual mean temperature is 10.4 °C^48^. There are few lakes within the system (7% lake area). The river is characterised by a high diversity of fish species, including salmonids (*Salmo trutta* and *Salmo salar*) as well as most of the species present in Möckeln (e.g. pike, pikeperch, perch, bream, silver bream and roach).

### Sampling procedures

With the aim to capture the complete size structure of the focal populations we sampled individuals in both systems (Lake Möckeln and River Emån) between May and September using two methods, longlining for targeting adults (> 70 cm) and fyke-nets for juveniles and subadults. Longlines consisted of a floating mainline (100–400 m depending on the sampled area) to which monofilament (1.2 mm) leaders (1–1.5 m, looped to Scotty release clips) were fitted with single treble hooks and baited with native cyprinids were attached every 10–20 m. Fyke-nets varied in design/size between systems, in Lake Möckeln we employed a large two-armed fyke-net (20–30 m arms, 1.5 m diameter entrance) whereas in River Emån we used a smaller single-arm fyke net (8 m arm, 0.5 m diameter entrance). Both fyke-nets and longlines were initially set randomly throughout the systems but over time placed in hot-spot locations to obtain higher n-values. All gear were checked daily.

All fish were measured (total length) and weighted immediately after capture and date of capture was recorded. Using a measuring tape, small individuals (< 40 cm) was measured to the nearest mm whereas the length of larger fish was determined to the closest cm. The weight of small individuals was determined to nearest gram (Berkley 50 lbs/22 kg), larger individuals to the nearest 0.1 kg (Steinberg Systems SBS-KW-300/100-O). All individuals were marked with a PIT-tag (Passive Integrated Transponder from Biomark, 23 mm HDX), injected in the pelvic girdle or the abdominal cavity. Recaptured individuals, as identified by the PIT-id, was measured and weighted to be able to compare growth since the initial day of tagging before released. The sample procedure of a catfish took < 5 min.

### Growth trajectories, age determination and statistics

The mark-recapture data was used to construct a von Bertalanffy growth curve (VBGC) of age at length by adopting the method by Fabens 1965^49^ (sensu Hamel 2014^15^). A reformulated VBGC-equation (ΔL = (L_∞_ − L_m_)(1 − e^(−K × ΔT)^) was fitted to the mark-recapture data of growth between mark and recapture (ΔL), number of years between mark and recapture (ΔT, fractional values) and length at tagging (L_m_). The parameters K and L_∞_ for the VBGC-equation were estimated iteratively by non-linear regression using the nls-function in the package *stats* (version 4.1.2, part of base R) in R (version 4.1.2; R Core Team^50^). Only individuals recaptured 10 months or more after tagging were included (*N* = 162) to ensure that all individuals had experienced at least one growth season. The last recapture with the longest time span between mark and recapture was used for individuals with repeated recaptures such that every individual contributed with one observation of growth. Length at hatching (0.7 cm^51^) was used as L0 in the VBGC-equation. A reformulated VBGC-equation (Age = log_e_ ((Length − L_∞_)/(L0 − L_∞_))/ − K) with the L0 and the estimated parameters K and L_∞_ was used to predict age at length.

The length (L) at weight (W) relationship was analysed by fitting a classic length–weight model with a power function (W = a × L^b^). The parameters a and b were estimated by fitting a linear regression of log_e_(W) as a function of log_e_(L).

### Ethics statement

Ethical approval for the study was granted by the Ethical Committee on Animal Research in Linköping, Sweden (approval Dnr 16867-2018). All methods were performed in accordance with relevant guidelines and regulations and the study is reported following the recommendations in the ARRIVE guidelines^52^.

## Data Availability

Data supporting the results is uploaded on www.datadryad.org (10.5061/dryad.qz612jmhs).
