# Author Correction: Exceptional longevity in northern peripheral populations of Wels catfish (*Siluris glanis*)

**DOI:** 10.1038/s41598-022-14484-4

**Published:** 2022-06-13

**Authors:** Kristofer Bergström, Oscar Nordahl, Peter Söderling, Per Koch‑Schmidt, Tobias Borger, Petter Tibblin, Per Larsson

**Affiliations:** 1 Department of Biology and Environmental Science, Centre for Ecology and Evolution in Microbial Model Systems EEMiS, Linnaeus University, 391 82 Kalmar, Sweden; 2 Fish and Wildlife, The County Administrative Board of Kalmar County, Regeringsgatan 1, 391 86 Kalmar, Sweden

Correction to: *Scientific Reports* https://doi.org/10.1038/s41598-022-12165-w, published online 16 May 2022

The original version of this Article contained a repeated error where *Silurus glanis* was incorrectly written as *Siluris glanis* in the title,

“Exceptional longevity in northern peripheral populations of Wels catfish (*Siluris glanis*)”

now reads:

“Exceptional longevity in northern peripheral populations of Wels catfish (*Silurus glanis*)”

In the Introduction,

“Here, we investigate growth trajectories and longevity in northern peripheral populations of Wels catfish (*Siluris glanis*, hereafter catfish).”

now reads:

“Here, we investigate growth trajectories and longevity in northern peripheral populations of Wels catfish (*Silurus glanis*, hereafter catfish).”

In the Reference list,

17. IUCN (International Union for Conservation of Nature) 2008. *Siluris glanis*. *The IUCN Red List of Threatened Species. Version 2021–3* (2010). https://www.iucnredlist.org. (Accessed 25 February 2021).

33. Alp, A., Kara, C. & Büyükcapar, H. M. Reproductive biology in a Native European Catfish, *Siluris glanis* L., 1758*,* population in Menzelet Resevoir. *Turk. J. Vet. Ani. Sci.* **28**, 613 (2004).

now reads:

17. IUCN (International Union for Conservation of Nature) 2008. *Silurus glanis*. *The IUCN Red List of Threatened Species. Version 2021–3* (2010). https://www.iucnredlist.org. (Accessed 25 February 2021).

33. Alp, A., Kara, C. & Büyükcapar, H. M. Reproductive biology in a Native European Catfish, *Silurus glanis* L., 1758*,* population in Menzelet Resevoir. *Turk. J. Vet. Ani. Sci.* **28**, 613 (2004).

The original Article has been corrected.

